# Application of Magnetic Adaptive Testing for Nondestructive Investigation of 2507 Duplex Stainless Steel

**DOI:** 10.3390/s23073702

**Published:** 2023-04-03

**Authors:** Gábor Vértesy, István Mészáros, Bálint Bögre

**Affiliations:** 1 Centre for Energy Research, 1121 Budapest, Hungary; 2 Department of Materials Science and Engineering, Faculty of Mechanical Engineering, Budapest University of Technology and Economics, 1111 Budapest, Hungary

**Keywords:** duplex stainless steel, magnetic adaptive testing, nondestructive magnetic testing, DC magnetometer

## Abstract

Duplex stainless steels are two-phase alloys, which contain ferritic and austenitic phases in their microstructure. Their duplex structure provides exceptional resistance to pitting and chloride stress corrosion cracking, and their strength is about twice that of austenitic stainless steels. Due to their good properties, they are widely used in chemical and petrochemical industries as a base material in pressure vessels, pipelines and containers. Duplex stainless steel samples were nondestructively investigated by measuring sets of magnetic minor hysteresis loops using the method called magnetic adaptive testing (MAT). Several series of heat-treated and cold-rolled 2507 duplex stainless steels were measured, and the magnetic parameters were compared with the results of the DC magnetometry of the samples. It was found that the changes in the material properties that were generated by heat treatment and mechanical deformation could easily be followed by magnetic measurements. In contrast to DC magnetic measurements, good correlation was found with the magnetic parameters determined by MAT method and Vickers hardness. Based on our experiments, MAT seems to be a powerful tool for the nondestructive characterization of duplex stainless steels.

## 1. Introduction

Duplex stainless steels are two-phase alloys, which contain ferrite and austenite phases in their microstructures [1,2,3]. This provides a combination of excellent corrosion resistance and greater strength. The strength of duplex stainless steels is about two times larger than that of austenitic stainless steels, and an improved resistance to localized corrosion is also observed. Compared to ferritic stainless steel, they also have improved toughness and ductility. This material is widely used in the chemical and petrochemical industries as a base material in pressure vessels, pipelines and containers.

One type of duplex stainless steel is 2507 (UNS S32750), which is a super duplex stainless steel designed for applications which require exceptional strength and corrosion resistance. It is an alloy of 25% chromium, 4% molybdenum and 7% nickel, which results in excellent resistance to chloride pitting and crevice corrosion attacks [4]. Its duplex structure provides 2507 with exceptional resistance to pitting and chloride stress corrosion cracking. In duplex stainless steels, several phase transformations can happen in the 300–1000 °C temperature range, generating segregation and the precipitation of new phases. A significant deterioration of the mechanical properties can be caused by the appearance of these phases [5,6]. The most significant phase transformation in duplex stainless steels is the eutectoidal decomposition of δ-ferrite while it transforms into the σ-phase and secondary austenite (δ→σ + γ_2_) [7]. The appearance of the σ-phase dramatically decreases the ductility of duplex stainless steel. Considering that these steels are widely used as construction materials, a nondestructive inspection of this process is of extremely great practical importance.

In contrast to destructive tests, nondestructive testing methods do not directly measure the mechanical properties of the investigated material, and before the practical application of any of them, their results must be carefully compared to the standardized destructive methods, such as—for instance—hardness measurements. Duplex stainless steels are ferromagnetic materials. This means that magnetic techniques can be used successfully for this purpose. Some magnetic properties are rather sensitive to the microstructure of the material, as described in [8]. The possible applications of magnetic methods in nondestructive evaluations are given in [9,10,11].

The effect of the sigma phase on the properties of duplex stainless steel was studied, verifying the detrimental effects of small percentages on corrosion resistance and toughness [7]. Saturation magnetization measurements were applied in a DC magnetometer to measure the amount of steel that was in the ferrite phase. These methods were found to be suitable to detect small percentages of the microstructure that were in the sigma phase.

A novel method of magnetic nondestructive testing has been developed recently. This method is called magnetic adaptive testing (MAT) [12]. With this technique, the investigated specimens are magnetized by a magnetizing yoke, and reliable parameters can be determined from the series of minor magnetic hysteresis loops. As an illustration of this method, similar samples to those studied in the present work (cold-rolled stainless steel) were measured [13].

It was demonstrated that magnetic quantities, which are closely related to the samples’ structural variation, main coercivity and remanence magnetization, can be determined much more sensitively from minor loops than from the major one. Consequently, the outcomes of analyses of the minor loops are more helpful than those of the conventional major loop measurements, and MAT is highly suitable for the sensitive and nondestructive characterization of structural changes in such materials. Another advantageous feature of this method is the confirmation that reliable parameters can be obtained by using the series of minor loops without magnetic saturation of the samples. Moreover, these measurements can be taken with a magnetizing yoke attached to the sample, and the yoke does not have to be special or large.

In a very recent work [14], the influence of heat treatment and that of plastic deformation were investigated upon the appearance of the sigma phase in 2507 duplex stainless steel. In this study, only the thermoelectric power measurements and magnetic saturation polarization were applied to monitor the microstructural changes generated by cold rolling and heat treatment. It was demonstrated that the magnetic saturation measurement and the thermo-electric power measurements were useful tools for monitoring the sigma-phase formation generated by heat input in 2507 duplex stainless steels. In the present work, the same sample set was investigated using MAT measurements in order to emphasize the capabilities and effectiveness of this method, compared to traditional magnetic measurements.

The purpose of the present work is to apply the MAT method to several series of cold-rolled and heat-treated 2507 duplex stainless steel, to compare the MAT results with the results of traditional DC magnetometry and also to compare the nondestructively determined magnetic parameters with the Vickers hardness of the samples.

## 2. Materials and Methods

### 2.1. Sample Preparation and Hardness Testing

Tests were performed on 2507-type steel. The nominal chemical composition of the tested AISI 2507 duplex stainless steel is shown in Table 1. The main alloying elements were chromium (about 25%) and nickel (about 7%).

The sample set was prepared in order to study the process of the eutectoidal phase transformation due to the previous cold rolling and heat treatment. Samples were cut from the sheet, as received, with a band saw. The initial *h*_0_ thickness of the samples was around 10 mm, the w_0_ width was around 15 mm and the *l*_0_ length of the cut samples was 100 mm. The specimens were cold-rolled by a double cylinder rolling machine with a 300 mm diameter. The cold rolling direction was perpendicular to the direction of the hot rolling during manufacturing. In every rolling step, the thickness reduction was 0.25 mm. 

**Figure 1:**
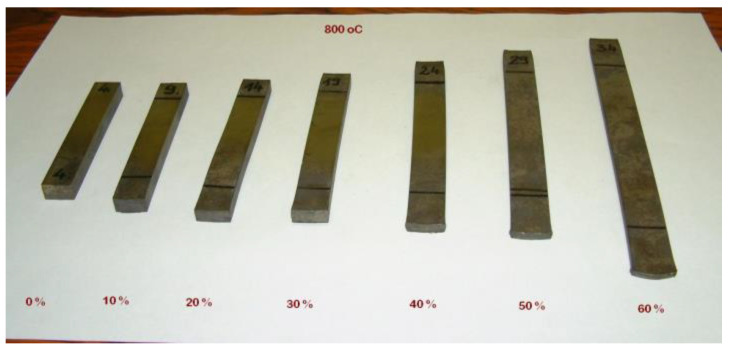
A specimen set. It contains an initial sample and six differently cold-rolled samples. All of them were heat-treated at 800 °C for 30 min.

The *ε* rolling reductions of the samples were the following: 0%, 10.3%, 22.3%, 31.3%, 41.6%, 50.6% and 61.9%. The rolling reductions were calculated using the following equation:

*ε* = (*h*_0_ − *h*)/*h*_0_ ∗ 100 (%) (1)

where *h* is the thickness of the rolled sample. From every rolling reduction, five samples were rolled, and they were heat-treated isothermally at temperatures of 20 °C, 700 °C, 750 °C, 800 °C and 850 °C. The time of the heat treatments was 30 min, and the samples were normalized in static normal air. Naturally, the different rolling reductions resulted in different sizes of samples (Figure 1).

The Vickers hardness of the specimens was measured by a KB 250 BVRZ-type hardness tester (KB Prüftechnik GmbH) with a load of 98.07 N.

### 2.2. DC Magnetometer Measurements

**Figure 2:**
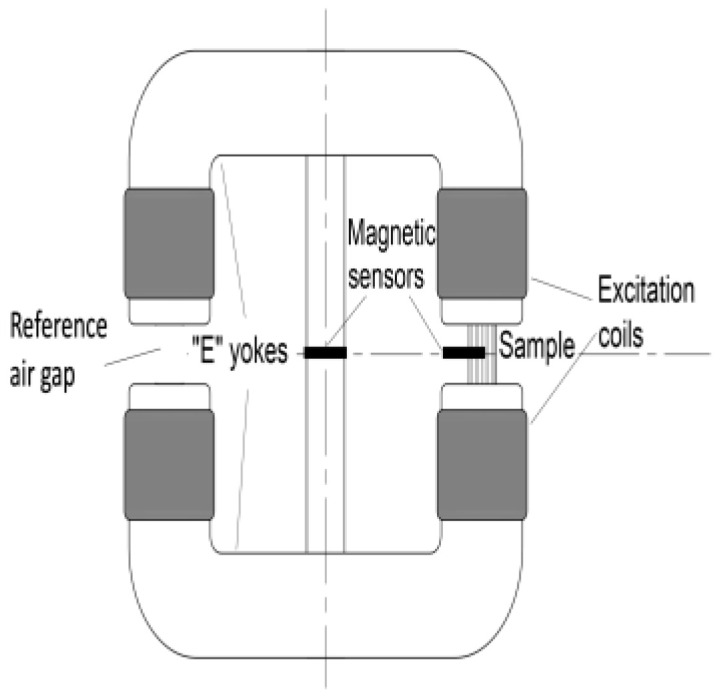
The setup of the applied Stablein-Steinitz DC magnetometer.

A Stablein-Steinitz DC magnetometer bridge was used for measuring the saturation magnetization loops, as shown in Figure 2. It has a symmetrical yoke with two U-shaped parts and a small cross-section in the middle bridge [15,16]. The arrangement has four excitation coils, two magnetic Hall sensors and two air gaps with uniform sizes (one for measuring and one for reference). If there is no sample in the measuring air gap, the set-up is magnetically symmetrical, and, consequently, there is no flux through the middle bridge. If a sample is fixed in the measuring air gap, the symmetry is broken. Consequently, some part of the magnetic flux closes through the middle bridge. The magnetic field measured by the Hall sensor in the middle bridge is directly proportional to the magnetization of the sample. The Stablein-Steinitz DC magnetometer is able to excite the bulk steel samples into magnetic saturation. It is one of the most precise methods of ferrite content measurement. The maximum excitation field strength is about 2700 A/cm.

### 2.3. Magnetic Adaptive Testing

A relatively new method called magnetic adaptive testing (MAT) has been developed for the nondestructive inspection of the degradation of structural materials. MAT is a special method of taking magnetic hysteresis measurements. The method is described in detail in [12]. During the measurement, minor magnetic hysteresis loops are systematically measured. The physical background of the measurement is that the modified parameters of the materials, caused by any type of degradation (plastic or elastic deformation, heat treatment, fatigue, corrosion, neutron irradiation, etc.), are reflected in the magnetic behaviour. The purpose of this technique is to obtain as much information as possible about the magnetic behaviours of the investigated samples and then to draw from the large data pool to pick up those parameters that characterize the actual material degradation the most sensitively and reliably. Different types of material degradations can be characterized by different parameters from this data pool. 

**Figure 3:**
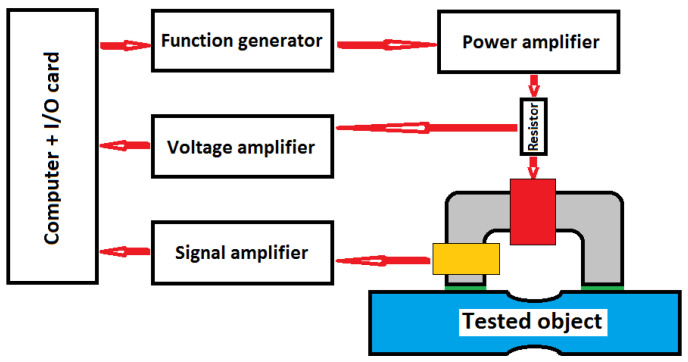
Block diagram of the MAT measuring system: The soft magnetic yoke (grey) magnetizes the blue tested object via the red driving coil (through the green non-magnetic thin spacers; this spacer is not necessary in all measurements) using a series of minor hysteresis loops with increasing amplitudes. The yellow sensing coil picks up the resulting signal, which carries information on the actual differential permeability, µ, of the closed magnetic circuit (and, thus, on the quality of the tested object). The amplitude at which the signal displays the top sensitivity with respect to the material quality of the object is singled out as the most responsive MAT measurement.

A block diagram of the MAT measuring system can be seen in Figure 3. Investigated specimens are magnetized with a magnetizing yoke, which is put on the flat surface of specimen. (Actually, in Figure 3 the surface of the specimen is not flat, but the same arrangement is applied to specimens with flat surfaces.) For this yoke, half of a transformer core is typically used. The material of which the yoke is composed is laminated Fe-Si sheets. The dimensions of the yoke depend on the sizes of the samples to be measured. The most important dimensions of the yoke are the cross section and the total outside length. In our experiments, they were 10 × 5 mm^2^, and 18 mm, respectively, corresponding to the sizes of the specimens. For magnetization, an excited coil, which was wound and placed on one of the legs of the yoke, was used. A linear and triangular waveform of the magnetizing field was applied with an amplitude that was increased step by step, generating one minor loop during each step. The magnetizing field that was pumped into the sample was proportional to the magnetizing current. 

**Figure 4:**
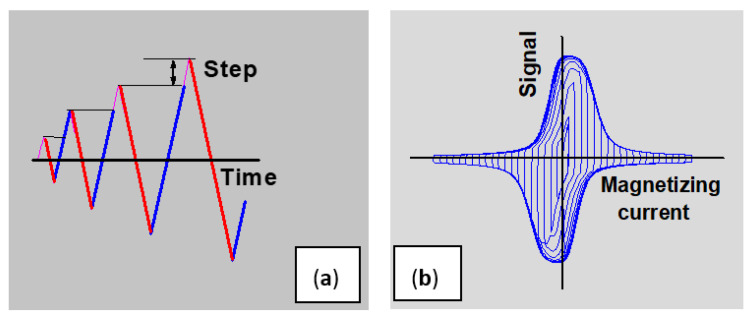
Time variation in magnetizing current (**a**) and the detected permeability loops (**b**) [17].

A pick-up coil, also wound around a yoke leg, was used for the measurement of the permeability generated by the triangularly increasing magnetizing field. The time variation in the magnetizing current and the detected permeability loops are shown in Figure 4. The variation of the magnetizing field was linear with time, which means that the pick-up coil’s signal was proportional to the differential permeability of the specimen. The slope of the magnetizing current in our experiment was 0.07 A/s. This slope had an influence on the value of the measured permeability, but it had no influence (within a certain range, of course) on the evaluated MAT descriptors. (In the same measurement series, the same value of slope must be applied.)

The measured permeability loops became the input data for any further data evaluation. Every point of these permeability loops provides information about the sample’s magnetic behaviour.

**Figure 5:**
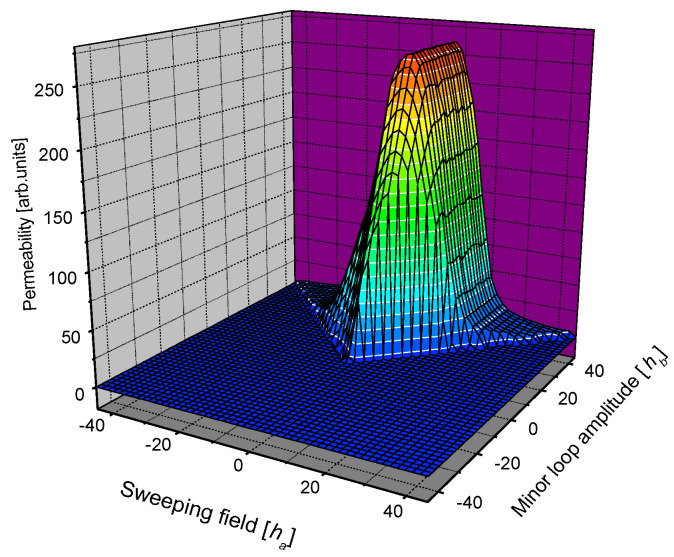
A 3D representation of the *m* permeability matrix.

Once the measured permeability loops were obtained, an evaluation process was performed. Instead of keeping the signal and the magnetizing field in shapes of continuous time-dependent functions, it was practical to interpolate the family of data for each sample into a discrete square matrix, *m* ≡ *m* (*h_a_*, *h_b_*), with a suitably chosen step, Δ*h_a_* = Δ*h_b_*. If the permeability values determined from the measured permeability loops are used, this is called a permeability, or *m*, matrix. (Other matrices can also be calculated, such as the hysteresis loops matrix, whose elements include the integrated permeability along the field, *h_a_*). A 3D representation of a permeability matrix is shown in Figure 5. The sweeping magnetizing field, *h_a_*, is given on the X axis and the amplitude of the corresponding minor loop, *h_b_*, is given on the Y axis, while the calculated permeability (from the measured loops) is given on the Z axis.

The *m* matrix elements were compared with the corresponding matrix element of the reference sample (as received). The modification of the normalized matrix elements as functions of the degradation parameter characterized the actual material degradations due to any external impact. A large data pool was calculated (with hundreds of matrix elements), and those descriptors which were the most sensitive to the actual material degradation were chosen from these matrix elements.

The possible sources of error of the MAT descriptors were carefully analyzed in [18]. It was found that the uncertainty of these descriptors was not more than 1%. 

In the present work, the value of the magnetizing current (A) is used instead of the field values (A/m) for the description of the magnetizing field, because this is a parameter that can be measured precisely. The real value of the magnetizing field inside each sample was not known due to the open magnetic circuit. Nevertheless, the magnetizing current was proportional to the magnetizing field, so this parameter was also suitable for the magnetic characterization of the measured material.

In general, magnetic parameters depend on the size of the investigated samples. Consequently, the results of measurements taken on samples of different sizes cannot be compared with each other. However, the sample size dependence of MAT descriptors was carefully analyzed in [19], and it was found that by choosing the proper size of magnetizing yoke, the degradation of the material could be correctly determined, even in the case of different sizes of samples. This was the situation in our case, and the size of the yoke was chosen to fit to the dimensions of the samples. The width of the yoke leg was 10 mm, and the width of each sample was about 15 mm (only slightly depending on the rolling reduction). The lengths of the samples were very large compared to their widths, so this parameter had no influence on the detected permeability loops. 

## 3. Results

**Figure 6:**
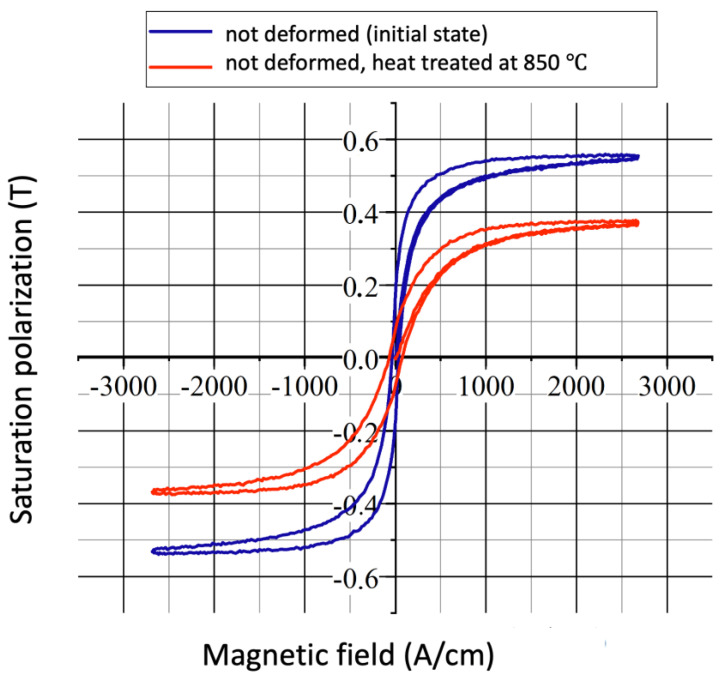
DC magnetization curves of undeformed heat-treated samples: saturation polarization vs. magnetic field.

All of the samples were measured using the two methods previously mentioned. The results of the DC magnetic measurements and magnetization curves of the undeformed heat-treated samples can be seen in Figure 6.

**Figure 7:**
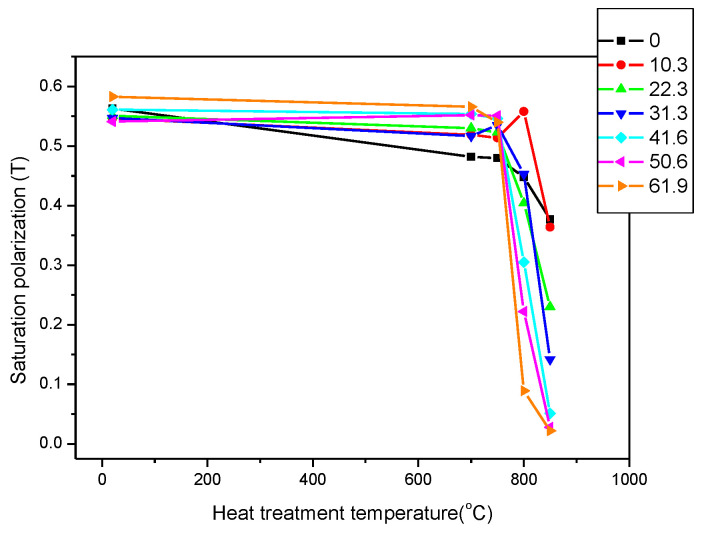
Saturation polarizations of all samples as a function of the temperature of the heat treatment. The parameter is the rolling reduction.

The saturation polarization is shown as a function of the temperature of the heat treatment in Figure 7. It clearly demonstrates that the magnetic parameters of unannealed (heat treatment temperature, T = 20 °C) samples in the figure were very close to each other, and even rolling reductions had no effect on their value. Heat treatments below 700 °C also had virtually no effect on the magnetic behaviour of the material, regardless of the rolling reduction. However, with heat treatments above 700 °C, the magnetic parameters started to decrease rapidly in all cases. Heat treatments at temperatures of 800 °C and 850 °C caused remarkable differences in the magnetic behaviours of the differently deformed samples.

**Figure 8:**
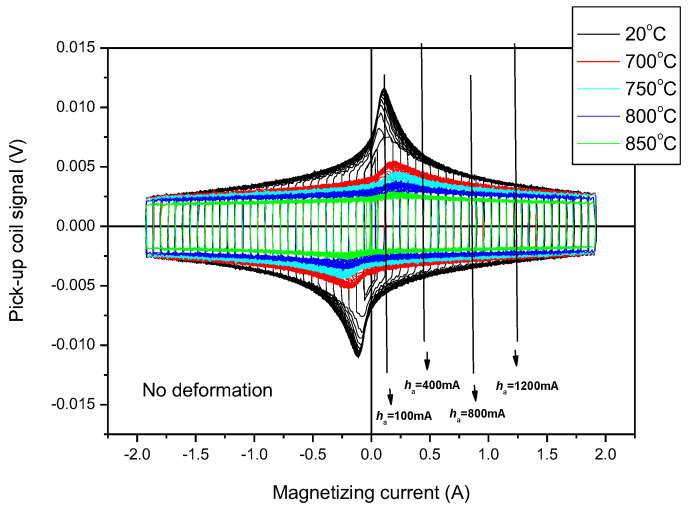
Measured permeability loops of undeformed heat-treated samples.

As an illustration of the MAT measurements, the series of measured permeability loops for samples without a rolling reduction is shown in Figure 8. The signal of the pick-up coil can be viewed as a function of the magnetizing current. (Minor loops are well-illustrated in the graphs.) The parameter is the temperature of the heat treatment. The influence of the heat treatments is reflected very well, especially if the maximal value of the permeability is considered. Some of the values of the magnetizing fields are marked, and an explanation can be found below.

**Figure 9:**
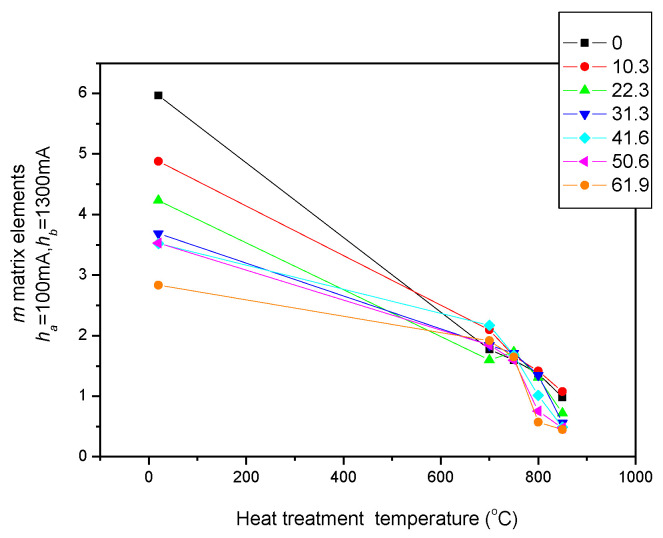
(*h_a_* = 100 and *h_b_* = 1300) MAT descriptors of all samples as a function of the temperature of heat treatments. The parameter is the rolling reduction.

The permeability loops illustrated above were used for calculating the *m* matrices. Considering that the most visible effect of the heat treatment is in the region where the magnetizing current is around *h_a_* = 100 mA (see Figure 8 at the first arrow, which is close to the maximal permeability of samples), *h_a_* = 100 mA MAT descriptors were calculated at the beginning. Figure 9 demonstrates how the MAT descriptors (*m* matrices) depended on the temperature of the heat treatments of the seven investigated series of samples, if the matrices were calculated using field values of *h_a_* = 100 mA and *h_b_* = 1300 mA. This MAT parameter offered the largest sensitivity.

In Figure 9, the influence of the heat treatments can be seen on the MAT descriptors (*h_a_* = 100 and *h_b_* = 1300) for the samples having rolling reductions from 0 to 61.9%. A field value of *h_a_* = 100 mA corresponds to the magnetizing field, where the maximal permeability was experienced (see Figure 8). The MAT descriptors of unannealed but differently cold-rolled samples differed significantly from each other. The heat treatments caused a significant reduction in this parameter. This behaviour is clearly different from that shown in Figure 7.

**Figure 10:**
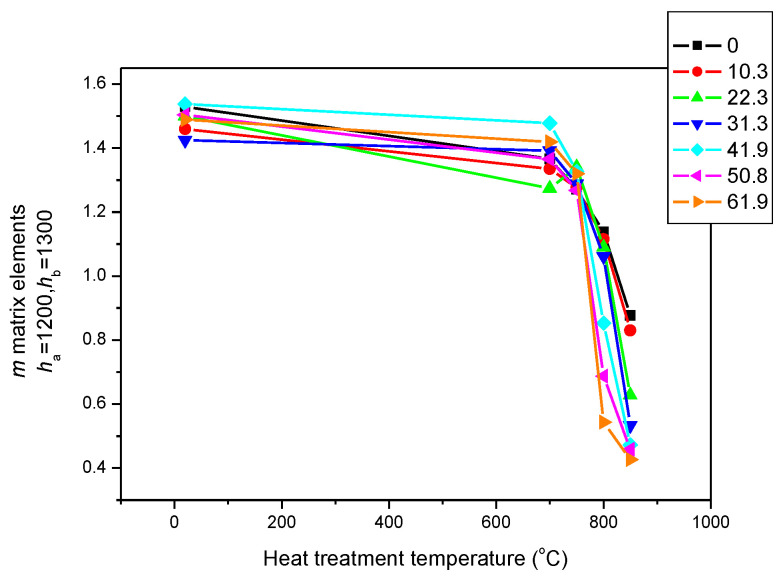
(*h_a_* = 1200, *h_b_* = 1300) MAT descriptors of all samples as a function of the temperature of the heat treatment. The parameter is the rolling reduction.

However, if another MAT descriptor (*h_a_* = 1200 and *h_b_* = 1300) is considered as a function of the temperature of the heat treatment (see Figure 10), another type of correlation can be observed, as shown in Figure 9. The sensitivity is smaller in this case, but the figure is more or less the same as Figure 7. The differences between the magnetic parameters belonging to the rolling reductions of 10.3 and 20.3 are attributed to measurement errors. 

All of this means that for the proper magnetic characterization of the heat treatment and the rolling reductions, the parameter of rolling reductions seems to be suitable. It is also marked in Figure 8, which is the region from which these parameters were taken. It is a feature of magnetic adaptive testing that the most suitable descriptor can be chosen from the large data pool that is generated.

**Figure 11:**
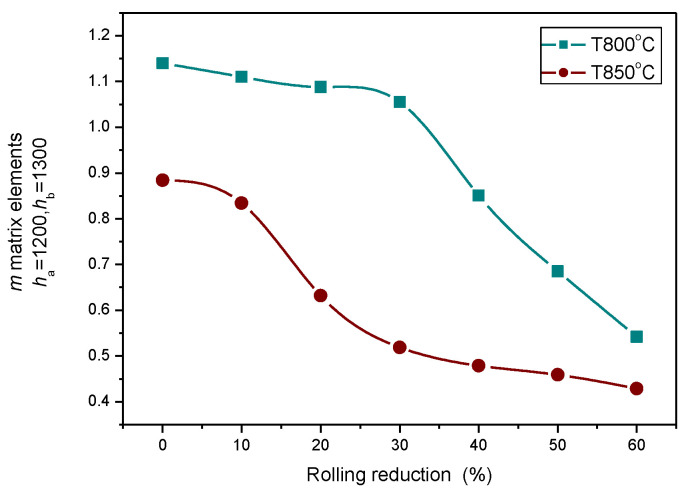
Modification of MAT descriptors as functions of rolling reductions for two annealing temperatures.

Heat treatments performed at 800 °C and 850 °C temperatures caused significant decreases in the magnetic parameters, as shown in Figure 11, and this modification depended very much on the rolling reduction. 

**Figure 12:**
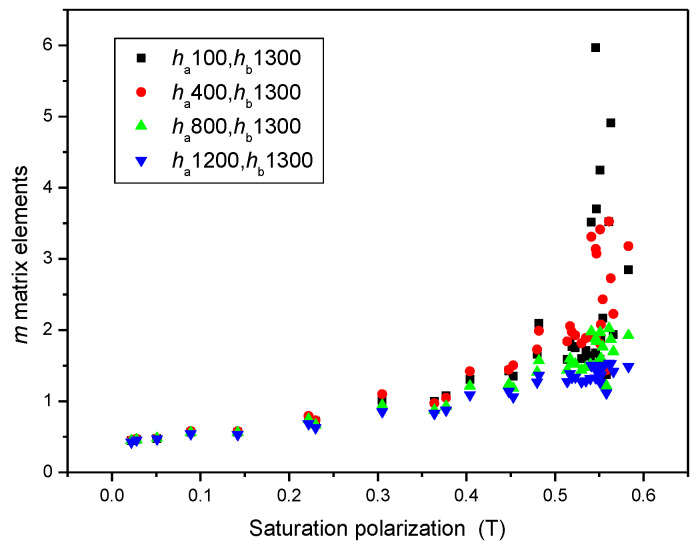
*m* matrix elements taken from four areas of permeability as functions of the saturation polarization. In this figure, all measured points are taken into account.

To prove that (*h_a_* = 1200 and *h_b_* = 1300) MAT descriptors were equal to the saturation induction measured by DC magnetometry, the correlation between MAT descriptors taken from different regions of permeability and saturation inductions is shown in Figure 12. It highlights that if the (*h_a_* = 1200 and *h_b_* = 1300) descriptors are considered, a very good and almost linear correlation exists between these two differently measured magnetic parameters, as shown by the blue triangles in Figure 12. In regions of lower magnetization, the difference becomes more and more pronounced, as shown by black squares in Figure 12. By taking MAT descriptors from the low magnetizing region, (*h_a_* = 100 and *h_b_* = 1300) the difference becomes significant (as demonstrated by the black squares).

**Figure 13:**
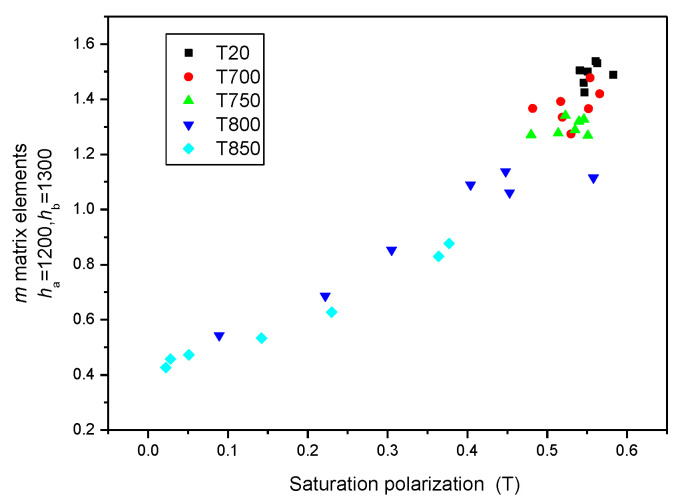
(*h*_a_ = 1200 and *h*_b_ = 1300) *m* matrix elements for all samples, indicating the actual heat treatments as functions of the saturation polarization.

Figure 13 is a magnification of the part of Figure 12 where only (*h_a_* = 1200 and *h_b_* = 1300) MAT descriptors are taken into account, but the different sample series are marked by different colours. The parameter in this figure is the temperature of the heat treatment. The equivalence of (*h_a_* = 1200 and *h_b_* = 1300) MAT parameters and the saturation induction is evident, regardless of the individual samples.

**Figure 14:**
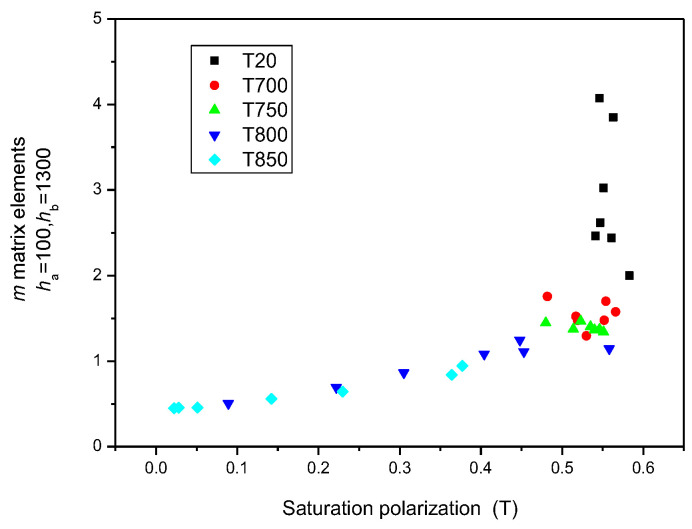
(*h*_a_ = 100 and *h*_b_ = 1300) *m* matrix elements for all samples, indicating the actual heat treatments as functions of the saturation polarization.

The situation becomes interesting if the (*h_a_* = 100 and *h_b_* = 1300) *m* matrix elements are considered by marking the different sample series again with different-coloured symbols. The correlation also seems to be good in this case, but only for those samples that were heat-treated. Samples without heat treatment (T = 20 °C) behaved very differently. These considerations are shown in Figure 14. 

**Figure 15:**
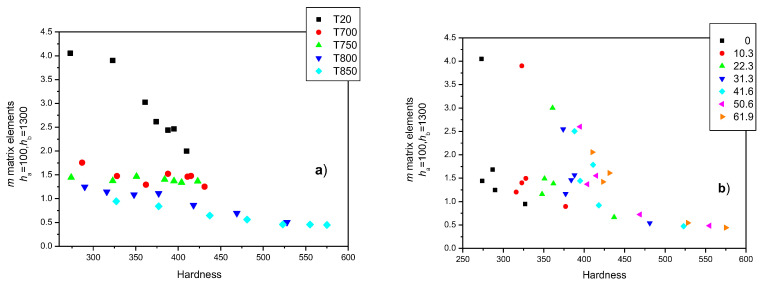
(*h*_a_ = 100 and *h*_b_ = 1300) *m* matrix elements for all samples, indicating the actual heat treatments as functions of the hardness (**a**) and indicating the actual rolling reductions (**b**).

MAT descriptors can also be considered as functions of the Vickers hardness. In this case, (*h_a_* = 100 and *h_b_* = 1300) *m* matrix elements were found to yield the best correlation between hardness and magnetic parameters. This correlation is shown in Figure 15. Interestingly, cold rolling causes a rapid and significant decrease in hardness if unannealed samples (the black squares) are considered, and there is an almost linear but less pronounced correlation between the magnetic parameters and the hardness due to cold rolling and heat treating at temperatures of 800 and 850 °C. Conversely, the magnetic parameters of the samples hardly seemed to be dependent on the hardness when heat treated at temperatures of 700 and 750 °C. As demonstrated in Figure 15a,b, the actual temperature value of the heat treatment seems to be important (Figure 15a) rather than the value of deformation (Figure 15b). 

**Figure 16:**
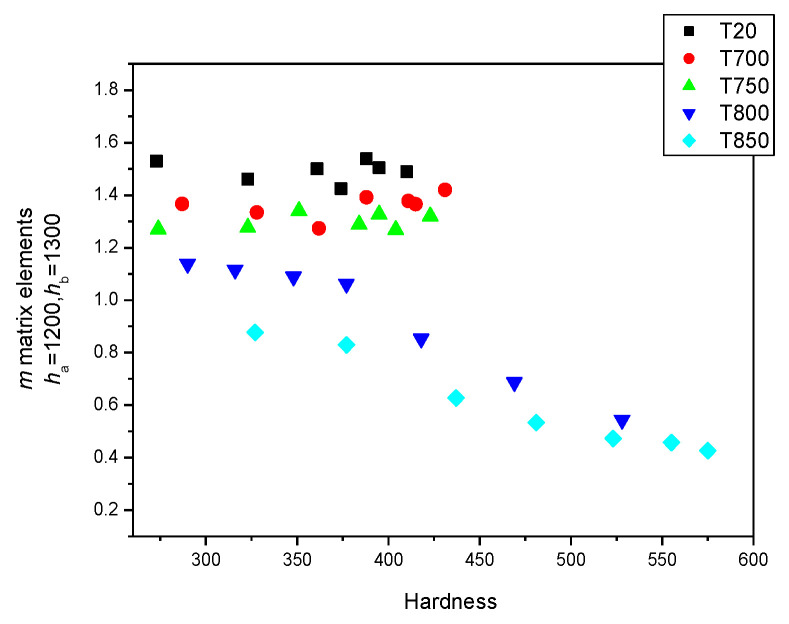
(*h*_a_ = 1200 and *h*_b_ = 1300) *m* matrix elements for all samples, indicating the actual heat treatments as functions of the hardness.

However, if the other group of magnetic descriptors (*h_a_* = 1200 and *h_b_* = 1300) are used for the magnetic characterization of the material, the correlation with the hardness is different, as shown in Figure 16. The difference in the MAT parameters vs. the hardness values is significant in the cases of deformed but unannealed samples.

**Figure 17:**
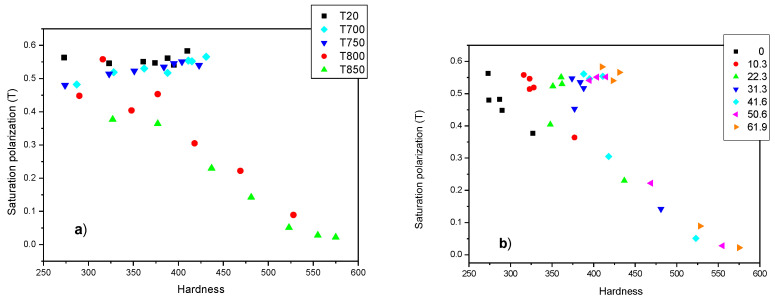
Saturation polarizations of all samples, indicating the actual heat treatments (**a**) and the rolling reductions (**b**) as functions of the hardness.

It is also possible to consider the saturation polarization as a function of the hardness, as shown in Figure 17. The correlation, or lack of correlation, is identical with the case of the (*h_a_* = 1200 and *h_b_* = 1300) *m* matrix elements.

**Figure 18:**
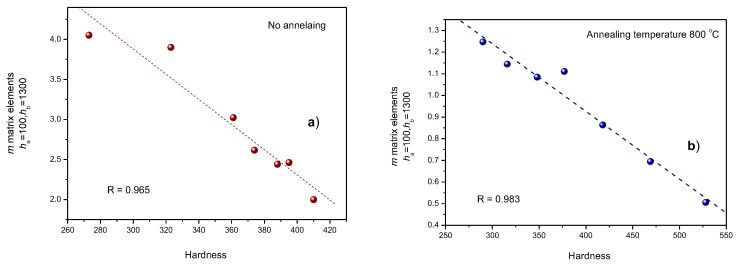
(*h*_a_ = 100 and *h*_b_ = 1300) MAT descriptors as functions of the hardness if the two groups of samples are considered separately, as follows: cold-rolled but unannealed samples (**a**) and samples that are cold-rolled and annealed at a temperature of 800 °C (**b**).

The good correlation between (*h_a_* = 100 and *h_b_* = 1300) *m* matrix elements are demonstrated better, if—for illustration—the two groups of samples are considered separately. This is illustrated in Figure 18, where the MAT descriptors are shown for cold-rolled but unannealed samples (Figure 18a) and for cold-rolled samples annealed at a temperature of 800 °C (Figure 18b). The regression factors of the linear fit are also indicated in the graphs. 

## 4. Discussion

Several series of cold-rolled and heat-treated 2507 duplex stainless steel samples were magnetically characterized using two techniques: DC magnetic measurements and MAT. It was found that each method was sensitive either to heat treatment or to cold rolling. If one group of MAT descriptors was considered, it was identical with the results of the DC measurements. 

This is a good result from the point of view of the magnetic characterization of materials; the different methods of taking measurements are in good correlation with each other. The Stablein-Steinitz DC magnetometer bridge is a frequently used and accepted means of taking magnetic measurements. The good correlation is a kind of validation of the MAT method as well. The DC magnetometry results in absolute values of magnetic quantities, in contrast to MAT, which only gives relative values, and it is only suitable for comparative measurements. On the other hand, MAT measurements can be performed on large-sized and irregularly shaped specimens as well, in contrast to the Stablein-Steinitz DC magnetometer bridge. Thus, only MAT can be considered only to be a true nondestructive testing method. 

Another advantage of MAT is that it offers many parameters which can give a more complex characterization of the material degradation. In the experiments described in this work, if the other group of MAT descriptors was taken into consideration, the correlation between the DC measurement and the MAT parameters was no longer as good. These MAT descriptors showed that, in terms of magnetization, the samples seemed to behave differently than indicated by DC magnetometry.

The principal difference in permeability is evident, as shown in Figure 8; it cannot be a measurement error. The repeatability of the measurement is excellent. The different behaviours of deformed but unannealed samples were not seen in the DC magnetic measurements, but if the “proper” descriptors were used, they were evident in the case of MAT. The saturation polarization was independent of the effect of the plastic deformation in the investigated duplex stainless steel. Some MAT parameters, on the other hand, were highly sensitive to the structure, so the magnetic hardening caused by the plastic deformation influenced them. This behaviour of duplex steels needs further discussion/investigation, as our measurements only call attention to this anomalous characteristic. 

The second group of MAT descriptors, calculated from the low magnetizing field region, does well at characterizing the modified mechanical hardness due to the heat treatment and the rolling reduction, in contrast to the results of the DC magnetic measurements. The good correlation between the MAT parameters and the hardness was also found in several other materials, such as [20]. In these cases, the good correlation was also found in the low magnetizing field region of permeability. 

## 5. Conclusions

In the present work, the same sample set, as measured by traditional magnetometry, was investigated using MAT measurements in order to emphasize the capabilities and effectiveness of this novel method, compared to traditional magnetic measurements. It was found that the changes in material properties that were generated by heat treatments and mechanical deformations could easily be followed by both types of magnetic measurement. DC magnetometry and MAT produced similar results. This fact can be considered to be a kind of validation of the MAT method.

However, in contrast to the DC magnetic measurements, a good correlation was also found between the MAT descriptors and the Vickers hardness, demonstrating the capability of MAT. Our experiments proved another advantage of the MAT method, which is that many parameters can be chosen from the large data pool that is generated, and different parameters can be used for the complex characterization of a given material. 

Last but not least, we would like to emphasize that, based on our experiments, MAT seems to be a powerful tool for nondestructive characterizations of structural elements of machinery that is composed of duplex stainless steels. The Stablein-Steinitz DC magnetometer cannot be considered to be a true nondestructive technique, because in contrast to MAT, it cannot measure large or irregular shape samples. 

## Figures and Tables

**Table 1:** Nominal chemical composition of the investigated AISI 2507 DSS material.

Fe	C	Mn	S	P	Si	Cu	Ni	Cr	Mo	Nb	N	Ti
Rest.	0.021	0.822	0.0004	0.023	0.313	0.178	6.592	24.792	3.705	0.008	0.264	0.005

## Data Availability

Not applicable.
